# Dichlorido{2-[2-(piperidin-1-yl)ethyl­imino­meth­yl]phenolato}zinc(II) monohydrate

**DOI:** 10.1107/S1600536808014311

**Published:** 2008-05-17

**Authors:** Dong-Fang Zhang, Mei-Huan Zhou, Chang-Ji Yuan

**Affiliations:** a School of Pharmaceutical Sciences, China Medical University, Shenyang 110001, People’s Republic of China; b Northeast Pharmaceutical Group Co. Ltd, Shenyang 110026, People’s Republic of China

## Abstract

In the title mononuclear zinc(II) complex, [ZnCl_2_(C_14_H_20_N_2_O)]·H_2_O, the Zn^II^ atom is four-coordinated by the phenolate O and imine N atoms of the Schiff base ligand and by two Cl atoms in a tetra­hedral geometry. In the crystal structure, O—H⋯Cl, O—H⋯O and N—H⋯O hydrogen bonds involving the water mol­ecules bridge adjacent complexes into a ladder-like structure running along the *c* axis.

## Related literature

For general background on Schiff base complexes, see: Kawamoto *et al.* (2008 ▶); Tomat *et al.* (2007 ▶). For biological properties of Schiff base compounds, see: Abd-Elzaher (2004 ▶); Iqbal *et al.* (2005 ▶); Osowole *et al.* (2005 ▶); Raman & Thangaraja (2005 ▶). For related structures, see: Ali *et al.* (2008 ▶); Li (2007 ▶); Tatar *et al.* (2002 ▶); Wang (2007 ▶).
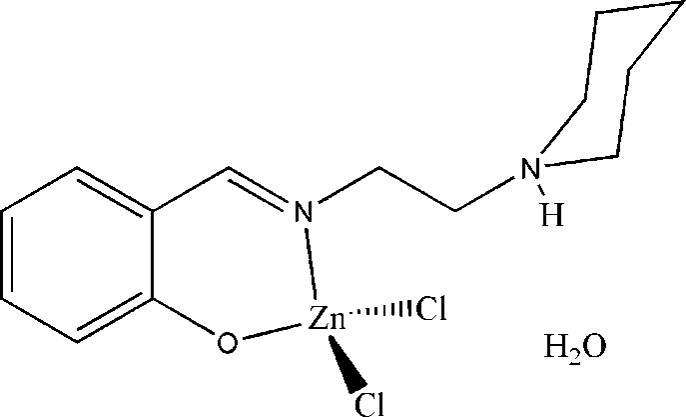


## Experimental

### 

#### Crystal data

[ZnCl_2_(C_14_H_20_N_2_O)]·H_2_O
*M* *_r_* = 386.61
Monoclinic, 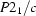

*a* = 9.1860 (18) Å
*b* = 19.875 (4) Å
*c* = 9.966 (2) Å
β = 110.20 (3)°
*V* = 1707.6 (7) Å^3^
*Z* = 4
Mo *K*α radiation
μ = 1.76 mm^−1^
*T* = 298 (2) K
0.20 × 0.18 × 0.17 mm

#### Data collection

Bruker SMART CCD area-detector diffractometer
Absorption correction: multi-scan (*SADABS*; Bruker, 2000 ▶) *T*_min_ = 0.720, *T*_max_ = 0.754
14151 measured reflections
3882 independent reflections
2685 reflections with *I* > 2σ(*I*)
*R*_int_ = 0.056

#### Refinement

*R*[*F* ^2^ > 2σ(*F* ^2^)] = 0.045
*wR*(*F* ^2^) = 0.104
*S* = 0.97
3882 reflections
199 parameters
4 restraints
H atoms treated by a mixture of independent and constrained refinement
Δρ_max_ = 0.49 e Å^−3^
Δρ_min_ = −0.58 e Å^−3^

### 

Data collection: *SMART* (Bruker, 2000 ▶); cell refinement: *SAINT* (Bruker, 2000 ▶); data reduction: *SAINT*; program(s) used to solve structure: *SHELXTL* (Sheldrick, 2008 ▶); program(s) used to refine structure: *SHELXTL*; molecular graphics: *SHELXTL*; software used to prepare material for publication: *SHELXTL*.

## Supplementary Material

Crystal structure: contains datablocks global, I. DOI: 10.1107/S1600536808014311/ci2598sup1.cif

Structure factors: contains datablocks I. DOI: 10.1107/S1600536808014311/ci2598Isup2.hkl

Additional supplementary materials:  crystallographic information; 3D view; checkCIF report

## Figures and Tables

**Table:** 

Zn1—O1	1.929 (2)
Zn1—N1	2.024 (2)
Zn1—Cl2	2.2066 (10)
Zn1—Cl1	2.2523 (10)

**Table:** 

O1—Zn1—N1	95.83 (10)
O1—Zn1—Cl2	113.80 (8)
N1—Zn1—Cl2	111.04 (8)
O1—Zn1—Cl1	109.48 (8)
N1—Zn1—Cl1	109.22 (8)
Cl2—Zn1—Cl1	115.66 (4)

**Table 2:** Hydrogen-bond geometry (Å, °)

*D*—H⋯*A*	*D*—H	H⋯*A*	*D*⋯*A*	*D*—H⋯*A*
N2—H2*C*⋯O2	0.90 (4)	1.81 (4)	2.712 (3)	177 (4)
O2—H2*B*⋯O1^i^	0.84 (3)	1.91 (3)	2.741 (3)	168 (4)
O2—H2*A*⋯Cl1^ii^	0.85 (3)	2.44 (3)	3.272 (3)	168 (4)

Symmetry codes: (i) 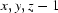; (ii) 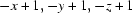.

**Fig. 1.**
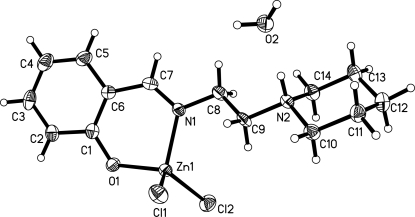
The molecular structure of the title compound, showing 30% probability displacement ellipsoids.

**Fig. 2.**
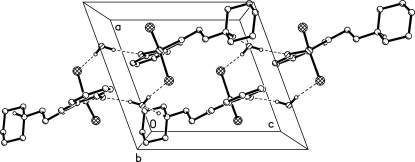
Crystal packing of the title compound. Intermolecular hydrogen bonds are shown as dashed lines. H atoms not involved in the interactions have been omitted for clarity.

## supplementary crystallographic information

### Comment

Zinc(II) complexes with Schiff base ligands have received much attention in recent years (Tomat *et al.*, 2007; Kawamoto *et al.*, 2008). Some of the complexes have been found to have biological properties (Osowole *et al.*, 2005; Iqbal *et al.*, 2005; Raman & Thangaraja, 2005; Abd-Elzaher, 2004). In this paper, the crystal structure of the title new zinc(II) complex with the Schiff base ligand 2-[(2-piperidin-1-ylethylimino)methyl]phenol is reported.

The title compound consists of a mononuclear Schiff base zinc(II) complex molecule and a water of hydration (Fig. 1). The Zn^II^ atom in the complex is four-coordinate in a tetrahedral geometry with one phenolate O and one imine N atoms of the Schiff base ligand, and with two Cl atoms. Bond lengths and angles (Table 1) about the Zn^II^ centre are comparable with the values observed in other Schiff base zinc(II) complexes (Wang, 2007; Ali *et al.*, 2008; Li, 2007; Tatar *et al.*, 2002). The crystal structure is stabilized by intermolecular O–H···Cl, O–H···O and N—H···O hydrogen bonds (Table 2 and Fig. 2).

### Experimental

A mixture of salicylaldehyde (0.1 mmol, 12.2 mg), 2-piperidin-1-ylethylamine (0.1 mmol, 12.8 mg) and ZnCl_2_ (0.1 mmol, 13.6 mg) in methanol was stirred for 30 min at room temperature to give a yellow solution. After keeping the solution in air for 12 d, yellow block-shaped crystals were formed.

### Refinement

Atoms H2A, H2B and H2C were located from a difference Fourier map and refined isotropically, with O-H, N-H, and H···H distances restrained to 0.85 (1), 0.90 (1), and 1.37 (2) Å, respectively. The remaining H atoms were placed in calculated positions and constrained to ride on their parent atoms, with C-H = 0.93 or 0.97 Å and *U*_iso_(H) = 1.2*U*_eq_(C).

### Crystal data

**Table:**

[ZnCl_2_(C_14_H_20_N_2_O)]·H_2_O	*F*_000_ = 800
*M**_r_* = 386.61	*D*_x_ = 1.504 Mg m^−^^3^
Monoclinic, *P*2_1_/*c*	Mo *K*α radiation λ = 0.71073 Å
Hall symbol: -P 2ybc	Cell parameters from 2461 reflections
*a* = 9.1860 (18) Å	θ = 2.4–25.0º
*b* = 19.875 (4) Å	µ = 1.76 mm^−^^1^
*c* = 9.966 (2) Å	*T* = 298 (2) K
β = 110.20 (3)º	Block, yellow
*V* = 1707.6 (7) Å^3^	0.20 × 0.18 × 0.17 mm
*Z* = 4	

### Data collection

**Table:**

Bruker SMART CCD area-detector diffractometer	3882 independent reflections
Radiation source: fine-focus sealed tube	2685 reflections with *I* > 2σ(*I*)
Monochromator: graphite	*R*_int_ = 0.056
*T* = 298(2) K	θ_max_ = 27.5º
ω scans	θ_min_ = 2.1º
Absorption correction: multi-scan(SADABS; Bruker, 2000)	*h* = −11→11
*T*_min_ = 0.720, *T*_max_ = 0.755	*k* = −25→25
14151 measured reflections	*l* = −12→12

### Refinement

**Table:**

Refinement on *F*^2^	Secondary atom site location: difference Fourier map
Least-squares matrix: full	Hydrogen site location: inferred from neighbouring sites
*R*[*F*^2^ > 2σ(*F*^2^)] = 0.045	H atoms treated by a mixture of independent and constrained refinement
*wR*(*F*^2^) = 0.104	*w* = 1/[σ^2^(*F*_o_^2^) + (0.0472*P*)^2^] where *P* = (*F*_o_^2^ + 2*F*_c_^2^)/3
*S* = 0.97	(Δ/σ)_max_ = 0.001
3882 reflections	Δρ_max_ = 0.49 e Å^−^^3^
199 parameters	Δρ_min_ = −0.58 e Å^−^^3^
4 restraints	Extinction correction: none
Primary atom site location: structure-invariant direct methods	

### Special details

**Table:**

Geometry. All e.s.d.'s (except the e.s.d. in the dihedral angle between two l.s. planes) are estimated using the full covariance matrix. The cell e.s.d.'s are taken into account individually in the estimation of e.s.d.'s in distances, angles and torsion angles; correlations between e.s.d.'s in cell parameters are only used when they are defined by crystal symmetry. An approximate (isotropic) treatment of cell e.s.d.'s is used for estimating e.s.d.'s involving l.s. planes.
Refinement. Refinement of *F*^2^ against ALL reflections. The weighted *R*-factor *wR* and goodness of fit *S* are based on *F*^2^, conventional *R*-factors *R* are based on *F*, with *F* set to zero for negative *F*^2^. The threshold expression of *F*^2^ > σ(*F*^2^) is used only for calculating *R*-factors(gt) *etc*. and is not relevant to the choice of reflections for refinement. *R*-factors based on *F*^2^ are statistically about twice as large as those based on *F*, and *R*- factors based on ALL data will be even larger.

### Fractional atomic coordinates and isotropic or equivalent isotropic displacement parameters (Å^2^)

**Table:**

	*x*	*y*	*z*	*U*_iso_*/*U*_eq_	
Zn1	0.30417 (4)	0.444609 (17)	0.67776 (4)	0.03531 (13)	
Cl1	0.53819 (11)	0.40618 (5)	0.68728 (10)	0.0547 (3)	
Cl2	0.13426 (12)	0.36691 (5)	0.68208 (10)	0.0566 (3)	
O1	0.3309 (3)	0.51454 (10)	0.8185 (2)	0.0466 (6)	
O2	0.2673 (3)	0.48851 (13)	0.0627 (3)	0.0540 (7)	
N1	0.2172 (3)	0.50529 (13)	0.5054 (2)	0.0330 (6)	
N2	0.1661 (3)	0.38887 (13)	0.1946 (3)	0.0322 (6)	
C6	0.2940 (3)	0.60705 (15)	0.6525 (3)	0.0349 (7)	
C1	0.3412 (4)	0.57904 (16)	0.7920 (3)	0.0360 (7)	
C2	0.3972 (4)	0.62356 (17)	0.9080 (3)	0.0423 (8)	
H2	0.4311	0.6063	1.0004	0.051*	
C3	0.4035 (4)	0.69155 (18)	0.8893 (4)	0.0494 (9)	
H3	0.4404	0.7197	0.9684	0.059*	
C4	0.3549 (4)	0.71867 (18)	0.7520 (4)	0.0553 (10)	
H4	0.3586	0.7649	0.7392	0.066*	
C5	0.3019 (4)	0.67719 (17)	0.6368 (4)	0.0481 (9)	
H5	0.2702	0.6956	0.5454	0.058*	
C7	0.2314 (4)	0.56952 (15)	0.5204 (3)	0.0351 (7)	
H7	0.1976	0.5948	0.4367	0.042*	
C8	0.1417 (4)	0.48009 (16)	0.3607 (3)	0.0420 (8)	
H8A	0.1519	0.5126	0.2919	0.050*	
H8B	0.0321	0.4730	0.3428	0.050*	
C9	0.2177 (4)	0.41441 (16)	0.3450 (3)	0.0379 (8)	
H9A	0.3293	0.4206	0.3789	0.045*	
H9B	0.1945	0.3807	0.4051	0.045*	
C10	0.2450 (4)	0.32330 (17)	0.1916 (3)	0.0414 (8)	
H10A	0.2136	0.2904	0.2482	0.050*	
H10B	0.3564	0.3292	0.2339	0.050*	
C11	0.2048 (4)	0.29752 (17)	0.0403 (3)	0.0454 (9)	
H11A	0.2538	0.2542	0.0419	0.054*	
H11B	0.2446	0.3284	−0.0141	0.054*	
C12	0.0304 (4)	0.29045 (18)	−0.0318 (4)	0.0507 (9)	
H12A	0.0064	0.2773	−0.1308	0.061*	
H12B	−0.0082	0.2558	0.0158	0.061*	
C13	−0.0471 (4)	0.35675 (18)	−0.0247 (3)	0.0476 (9)	
H13A	−0.1587	0.3514	−0.0667	0.057*	
H13B	−0.0156	0.3900	−0.0805	0.057*	
C14	−0.0063 (4)	0.38173 (17)	0.1267 (3)	0.0422 (8)	
H14A	−0.0555	0.4249	0.1267	0.051*	
H14B	−0.0442	0.3503	0.1814	0.051*	
H2C	0.198 (5)	0.4214 (14)	0.148 (4)	0.080*	
H2B	0.295 (4)	0.492 (2)	−0.009 (2)	0.080*	
H2A	0.330 (4)	0.5124 (18)	0.128 (3)	0.080*	

### Atomic displacement parameters (Å^2^)

**Table:**

	*U*^11^	*U*^22^	*U*^33^	*U*^12^	*U*^13^	*U*^23^
Zn1	0.0448 (3)	0.0329 (2)	0.0281 (2)	0.00015 (17)	0.01246 (17)	0.00067 (15)
Cl1	0.0473 (6)	0.0665 (6)	0.0490 (5)	0.0112 (5)	0.0150 (4)	0.0005 (5)
Cl2	0.0635 (6)	0.0507 (5)	0.0599 (6)	−0.0155 (5)	0.0266 (5)	0.0007 (4)
O1	0.0800 (18)	0.0341 (13)	0.0296 (12)	−0.0072 (12)	0.0238 (12)	−0.0028 (10)
O2	0.0692 (19)	0.0562 (16)	0.0411 (14)	−0.0195 (13)	0.0248 (14)	−0.0026 (12)
N1	0.0398 (16)	0.0353 (14)	0.0255 (13)	0.0008 (12)	0.0132 (12)	−0.0010 (11)
N2	0.0331 (15)	0.0381 (14)	0.0252 (13)	0.0002 (12)	0.0099 (11)	0.0004 (11)
C6	0.0319 (18)	0.0368 (17)	0.0374 (18)	0.0024 (14)	0.0138 (15)	0.0022 (14)
C1	0.0355 (19)	0.0385 (17)	0.0386 (19)	0.0004 (14)	0.0187 (15)	−0.0065 (14)
C2	0.046 (2)	0.047 (2)	0.0370 (19)	−0.0021 (16)	0.0183 (16)	−0.0113 (15)
C3	0.037 (2)	0.045 (2)	0.068 (3)	−0.0056 (16)	0.0200 (19)	−0.0248 (19)
C4	0.050 (2)	0.0339 (19)	0.080 (3)	0.0040 (17)	0.020 (2)	−0.0045 (19)
C5	0.049 (2)	0.0342 (18)	0.060 (2)	0.0049 (16)	0.0171 (19)	0.0042 (17)
C7	0.0336 (18)	0.0404 (19)	0.0336 (18)	0.0073 (14)	0.0145 (15)	0.0088 (14)
C8	0.047 (2)	0.048 (2)	0.0294 (17)	0.0098 (17)	0.0113 (16)	0.0019 (15)
C9	0.043 (2)	0.0495 (19)	0.0190 (16)	0.0072 (16)	0.0082 (14)	−0.0001 (13)
C10	0.039 (2)	0.0447 (19)	0.0378 (19)	0.0078 (16)	0.0102 (16)	0.0010 (15)
C11	0.045 (2)	0.045 (2)	0.045 (2)	0.0031 (16)	0.0135 (17)	−0.0076 (16)
C12	0.049 (2)	0.053 (2)	0.047 (2)	−0.0069 (18)	0.0124 (18)	−0.0116 (17)
C13	0.036 (2)	0.063 (2)	0.0363 (19)	−0.0028 (17)	0.0029 (16)	−0.0081 (17)
C14	0.0313 (19)	0.050 (2)	0.041 (2)	0.0048 (16)	0.0077 (15)	−0.0008 (16)

### Geometric parameters (Å, °)

**Table:**

Zn1—O1	1.929 (2)	C4—H4	0.93
Zn1—N1	2.024 (2)	C5—H5	0.93
Zn1—Cl2	2.2066 (10)	C7—H7	0.93
Zn1—Cl1	2.2523 (10)	C8—C9	1.514 (4)
O1—C1	1.319 (4)	C8—H8A	0.97
O2—H2B	0.84 (3)	C8—H8B	0.97
O2—H2A	0.85 (3)	C9—H9A	0.97
N1—C7	1.287 (4)	C9—H9B	0.97
N1—C8	1.457 (4)	C10—C11	1.512 (4)
N2—C9	1.496 (4)	C10—H10A	0.97
N2—C10	1.496 (4)	C10—H10B	0.97
N2—C14	1.499 (4)	C11—C12	1.520 (5)
N2—H2C	0.90 (4)	C11—H11A	0.97
C6—C5	1.407 (4)	C11—H11B	0.97
C6—C1	1.420 (4)	C12—C13	1.511 (5)
C6—C7	1.448 (4)	C12—H12A	0.97
C1—C2	1.404 (4)	C12—H12B	0.97
C2—C3	1.368 (5)	C13—C14	1.507 (4)
C2—H2	0.93	C13—H13A	0.97
C3—C4	1.393 (5)	C13—H13B	0.97
C3—H3	0.93	C14—H14A	0.97
C4—C5	1.360 (5)	C14—H14B	0.97
			
O1—Zn1—N1	95.83 (10)	C9—C8—H8A	110.0
O1—Zn1—Cl2	113.80 (8)	N1—C8—H8B	110.0
N1—Zn1—Cl2	111.04 (8)	C9—C8—H8B	110.0
O1—Zn1—Cl1	109.48 (8)	H8A—C8—H8B	108.3
N1—Zn1—Cl1	109.22 (8)	N2—C9—C8	113.5 (2)
Cl2—Zn1—Cl1	115.66 (4)	N2—C9—H9A	108.9
C1—O1—Zn1	123.71 (18)	C8—C9—H9A	108.9
H2B—O2—H2A	106 (2)	N2—C9—H9B	108.9
C7—N1—C8	116.8 (3)	C8—C9—H9B	108.9
C7—N1—Zn1	119.9 (2)	H9A—C9—H9B	107.7
C8—N1—Zn1	123.31 (19)	N2—C10—C11	111.2 (2)
C9—N2—C10	109.1 (2)	N2—C10—H10A	109.4
C9—N2—C14	113.8 (2)	C11—C10—H10A	109.4
C10—N2—C14	110.6 (2)	N2—C10—H10B	109.4
C9—N2—H2C	103 (3)	C11—C10—H10B	109.4
C10—N2—H2C	112 (3)	H10A—C10—H10B	108.0
C14—N2—H2C	108 (3)	C10—C11—C12	110.9 (3)
C5—C6—C1	119.1 (3)	C10—C11—H11A	109.4
C5—C6—C7	115.4 (3)	C12—C11—H11A	109.4
C1—C6—C7	125.4 (3)	C10—C11—H11B	109.4
O1—C1—C2	118.6 (3)	C12—C11—H11B	109.4
O1—C1—C6	123.9 (3)	H11A—C11—H11B	108.0
C2—C1—C6	117.4 (3)	C13—C12—C11	109.5 (3)
C3—C2—C1	122.1 (3)	C13—C12—H12A	109.8
C3—C2—H2	119.0	C11—C12—H12A	109.8
C1—C2—H2	119.0	C13—C12—H12B	109.8
C2—C3—C4	120.1 (3)	C11—C12—H12B	109.8
C2—C3—H3	120.0	H12A—C12—H12B	108.2
C4—C3—H3	120.0	C12—C13—C14	112.1 (3)
C5—C4—C3	119.7 (3)	C12—C13—H13A	109.2
C5—C4—H4	120.2	C14—C13—H13A	109.2
C3—C4—H4	120.2	C12—C13—H13B	109.2
C4—C5—C6	121.6 (3)	C14—C13—H13B	109.2
C4—C5—H5	119.2	H13A—C13—H13B	107.9
C6—C5—H5	119.2	N2—C14—C13	110.0 (3)
N1—C7—C6	127.5 (3)	N2—C14—H14A	109.7
N1—C7—H7	116.2	C13—C14—H14A	109.7
C6—C7—H7	116.2	N2—C14—H14B	109.7
N1—C8—C9	108.6 (2)	C13—C14—H14B	109.7
N1—C8—H8A	110.0	H14A—C14—H14B	108.2

### Hydrogen-bond geometry (Å, °)

**Table:**

*D*—H···*A*	*D*—H	H···*A*	*D*···*A*	*D*—H···*A*
N2—H2C···O2	0.90 (4)	1.81 (4)	2.712 (3)	177 (4)
O2—H2B···O1^i^	0.84 (3)	1.91 (3)	2.741 (3)	168 (4)
O2—H2A···Cl1^ii^	0.85 (3)	2.44 (3)	3.272 (3)	168 (4)

Symmetry codes: (i) *x*, *y*, *z*−1; (ii) −*x*+1, −*y*+1, −*z*+1.
